# In-Depth Two-Year Study of Phenolic Profile Variability among Olive Oils from Autochthonous and Mediterranean Varieties in Morocco, as Revealed by a LC-MS Chemometric Profiling Approach

**DOI:** 10.3390/ijms18010052

**Published:** 2016-12-28

**Authors:** Aadil Bajoub, Santiago Medina-Rodríguez, Lucía Olmo-García, El Amine Ajal, Romina P. Monasterio, Hafida Hanine, Alberto Fernández-Gutiérrez, Alegría Carrasco-Pancorbo

**Affiliations:** 1 Department of Analytical Chemistry, Faculty of Sciences, University of Granada, Ave. Fuentenueva, s/n, 18071 Granada, Spain; aliam80@hotmail.com (A.B.); smedina@ugr.es (S.M.-R.); luciaolmo@ugr.es (L.O.-G.); albertof@ugr.es (A.F.-G.); 2 Laboratory of Bioprocess and Bio-Interfaces, Faculty of Science and Technology, 23000 Beni Mellal, Morocco; hafidahanine0@gmail.com; 3 Provincial Department of Agriculture of Azilal, P.O. Box 13, 22000 Azilal, Morocco; amine_266@hotmail.com; 4 Instituto de Biología Agrícola de Mendoza (IBAM), CONICET. Alt. Brown 500, Chacras de Coria, 5505 Mendoza, Argentina; rmonasterio@mendoza-conicet.gob.ar

**Keywords:** olive oil, liquid chromatography-mass spectrometry, phenolic compounds, Moroccan varieties, Mediterranean varieties, varietal origin

## Abstract

Olive oil phenolic fraction considerably contributes to the sensory quality and nutritional value of this foodstuff. Herein, the phenolic fraction of 203 olive oil samples extracted from fruits of four autochthonous Moroccan cultivars (“Picholine Marocaine”, “Dahbia”, “Haouzia” and “Menara”), and nine Mediterranean varieties recently introduced in Morocco (“Arbequina”, “Arbosana”, “Cornicabra”, “Frantoio”, “Hojiblanca”, “Koroneiki”, “Manzanilla”, “Picholine de Languedoc” and “Picual”), were explored over two consecutive crop seasons (2012/2013 and 2013/2014) by using liquid chromatography-mass spectrometry. A total of 32 phenolic compounds (and quinic acid), belonging to five chemical classes (secoiridoids, simple phenols, flavonoids, lignans and phenolic acids) were identified and quantified. Phenolic profiling revealed that the determined phenolic compounds showed variety-dependent levels, being, at the same time, significantly affected by the crop season. Moreover, based on the obtained phenolic composition and chemometric linear discriminant analysis, statistical models were obtained allowing a very satisfactory classification and prediction of the varietal origin of the studied oils.

## 1. Introduction

Virgin olive oil (VOO) is a quite popular and nutritionally important and valuable vegetable oil, which is widely produced in the Mediterranean Basin and areas with similar climatic conditions for human consumption, but also with cosmetic and medicinal purposes [1]. Data from the International Olive Council (IOC) reveal that, to date, more than 11 million ha are devoted to *Olea europaea* L. cultivation all over the world, especially, in Mediterranean countries, where 98% of this area is concentrated [2].

Morocco represents the Southwestern extreme part of the Mediterranean olive tree landscape. In this country, olive cultivation and oil production are a rooted tradition, both as a income for more than 450,000 farmers as well as a high environmental value crop, due to its role in soil protection, particularly, in mountain farms [3]. Furthermore, over the last years, Moroccan olive area has remarkably increased from 763,000 ha in 2007/2008 to 1,020,000 ha in 2013/2014 [2,4]. Likewise, during the same period, Moroccan olive oil production has increased from 85,000 to 130,000 tons [5], making this crop as one of the most profitable and strategic horticulture crop in the country. However, despite this importance, the productivity of the olive sector in most Moroccan olive growing areas is still far below the local potential, showing a strong year-to-year fluctuation [6]. One of the main reasons is that olive cultivation in Morocco has traditionally been based on planting a single cultivar so-called “Picholine Marocaine” (occupying almost 90% of the total Moroccan olive cultivated area) [7]. This autochthonous cultivar shows good adaptability to a wide range of Moroccan pedoclimatic conditions, and exhibits interesting agronomic traits, oil quality, and composition characteristics [8]; however it presents some limitations, such as an accentuated alternate bearing [6] and susceptibility to the main olive fungal diseases, particularly those caused by *Spilocaea oleagina*, *Verticillium dahliae* and *Fusarium solani* [9]. Moreover, extensive research supports the high phenotypic and genetic variability of this cultivar in different Moroccan olive growing regions (almost in the same orchard), suggesting that the name of “Picholine Marocaine” encompass a pool of various local genotypes [10,11]. In the agricultural context, this fact can complicate the olive orchard management when nutrients, irrigation and other agricultural practices must be applied accordingly to the cultivar demands and its agronomic characteristics. Therefore, to cope with these constraints that can hinder the development of the olive oil sector in Morocco, and meet the current needs of Moroccan olive oil industry searching for more productive cultivars with specific traits, the National Institute of Agronomic Research has extensively worked on breeding programs, mainly based on clonal selection (which allowed getting some clonal selection genotypes, such as “Haouzia” and “Menara”) [12], cross-breeding [13], and comparative studies of agronomic and adaptive traits of some Mediterranean varieties recently introduced in Morocco [14].

Similarities and differences (in terms of morphological and agronomical characteristics) among promising selected Moroccan autochthonous and recently introduced olive varieties are, to date, well established [12,14,15]. Researches characterizing oils obtained from these varieties have mostly focused on their quality parameters evaluation, although some studies can be also found regarding genetic, environmental and technological factors influencing the quality of these oils, and their fatty acids and triacylglycerols composition [7,16,17,18,19,20,21]. However, to the best of our knowledge, there are no studies evaluating the nutritional value of these oils. Indeed, we can say that there is a knowledge gap concerning their composition and content on bioactive compounds, such as phenolic compounds, phytosterols and tocopherols; substances that are widely recognized as some of the most relevant components responsible of diverse nutritional and health beneficial properties of olive oil [22,23,24,25,26].

VOO contains a number of phenolic compounds which determine important sensory characteristics, such as astringency and bitterness [22,27]. These compounds and their various biological activities, such as antioxidant, antimicrobial, anti-inflammation, anti-aging, anticancer and cardio-protective properties have been associated with the beneficial effects of this oily matrix [28]. Besides, phenolic compounds have an important role in pattern recognition studies, being described as relevant markers for the varietal and geographical origin classification of VOO [29]. All the just mentioned reasons can explain the attention paid, over the last decades, by the scientific community to these type of compounds, focusing, for instance, on the improvement of the analytical methodologies to carry out their determination [30,31], the comprehensive characterization of their composition and content on VOOs produced from different varieties cultivated in various Mediterranean countries [32,33,34], and the monitoring of changes undergone on their composition as a result of intrinsic and extrinsic factors [35,36].

The aim of the present work was to thoroughly determine and compare, over two consecutive crop seasons (2012/2013 and 2013/2014), the phenolic profile of “Picholine Marocaine” VOOs (as the most widespread and typical Moroccan olive oil variety) with those of oils obtained from three Moroccan clonal selected varieties (“Dahbia”, “Haouzia” and “Menara”) and nine Mediterranean varieties recently introduced in Morocco (“Arbequina”, “Arbosana”, “Cornicabra”, “Frantoio”, “Hojiblanca”, “Koroneiki”, “Manzanilla”, “Picholine de Languedoc” and “Picual”). All the varieties were grown under identical pedoclimatic conditions in the same experimental olive orchard. Two liquid chromatography instruments were coupled to mass spectrometry (one of analyzers was of high MS resolution and the other one of low resolution), achieving, for the first time, qualitative and quantitative data on the phenolic pattern of the studied oils. The possible influence of the harvest year and variety on the content of identified phenolic compounds was obviously examined. Finally, the feasibility of combining phenolic compounds data and chemometrics (Principal component analysis (PCA) and Linear discriminant analysis (LDA)) to construct chemometric models able to trace the varietal origin of studied oils was investigated.

## 2. Results and Discussion

### 2.1. Phenolic Compounds Profiles Characterization

**Figure 1:**
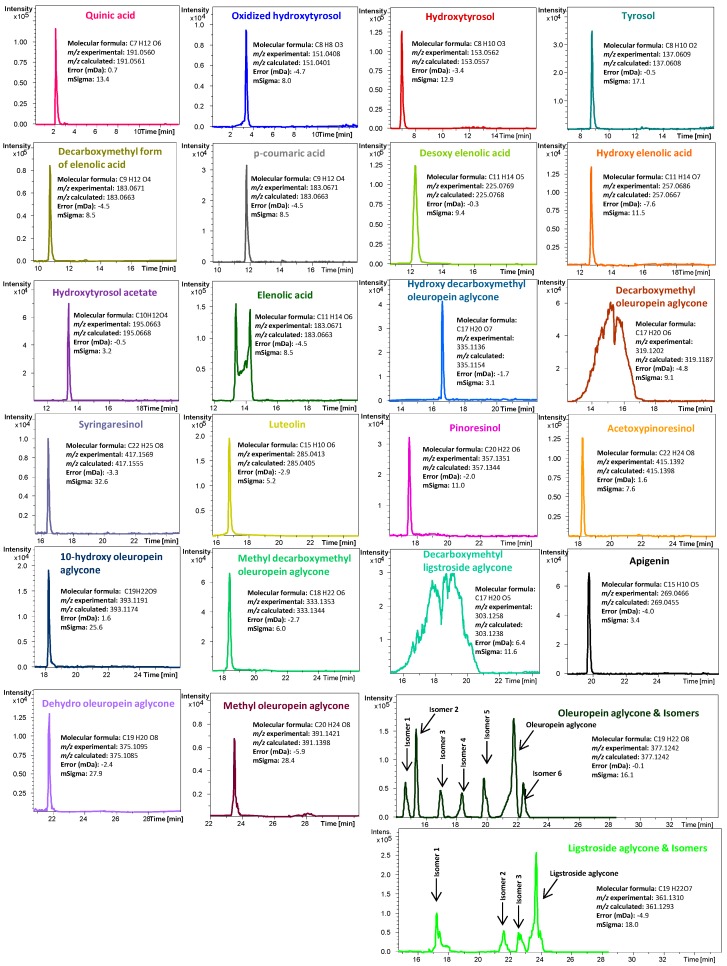
Extracted ion chromatograms (EICs) of the main phenolic compounds identified in the studied monovarietal olive oils, together with their MS data (molecular formula, experimental and calculated accurate mass to charge (*m/z*) signal, error, and mSigma value) obtained by liquid chromatography-electrospray ionization-time of flight mass spectrometry (LC-ESI-TOF MS).

Total ion chromatograms (TICs) and base peak chromatograms (BPC) achieved by the LC-ESI-TOF MS platform were explored and the extracted ion chromatograms (EICs) of detected phenolic compounds were depicted. Typical EICs of all the identified phenolic compounds are presented in Figure 1, together with their molecular formula, experimental and calculated mass to charge signal (*m/z*), error, mSigma value, and the proposed identity for each peak. Thus, as can be seen in this Figure, apart from quinic acid (QA) (a polar non-phenolic compound), up to 32 phenolic compounds and derivatives were tentatively identified in all the oils from the selected cultivars.

The phenolic compounds identified can be classified according to their chemical structure as follows: secoiridoids (22 compounds: 10-hydroxy oleuropein aglycone (10 Hy-Ol Agl), dialdehydic form of decarboxymethyl oleuropein aglycone (DOA), dehydro oleuropein aglycone (Dehydro Ol Agl), dialdehydic form of decarboxymethyl ligstroside aglycone (D-Lig Agl), desoxy elenolic acid (Desoxy-EA), dialdehydic form of decarboxymethyl elenolic acid (D-Ald-D EA), elenolic acid (EA), hydroxy decarboxymethyl oleuropein aglycone (Hy D-Ol Agl), hydroxy elenolic acid (Hy-EA), ligstroside aglycone (Lig Agl) and 3 isomers (Lig Agl IS1, Lig Agl IS2 and Lig Agl IS3), methyl decarboxymethyl oleuropein aglycone (Methyl D-Ol Agl), methyl oleuropein aglycone (Methyl Ol Agl), and oleuropein aglycone (Ol Agl) and 6 isomers (Ol Agl IS1, Ol Agl IS2, Ol Agl IS3, Ol Agl IS4, Ol Agl IS5 and Ol Agl IS1)); simple phenols (4 compounds: hydroxytyrosol (Hyt), hydroxytyrosol acetate (Hyt-Ac), oxidized hydroxytyrosol (O-Hyt) and tyrosol (Ty)); lignans (3 compounds: (+)-pinoresinol (Pin), (+)-1-acetoxypinoresinol (Ac-Pin) and syringaresinol (Syr)), flavonoids (2 compounds: luteolin (Lut) and apigenin (Apig)), and phenolic acids (*p*-coumaric acid (*p*-coum)).

The qualitative phenolic composition obtained herein is in good agreement with previously published reports where the phenolic fraction of monovarietal VOOs was evaluated [32,37]. The presence of multiple isomers of Ol Agl and Lig Agl is something that we would like to clarify before continuing. Since the publication of two very complete papers written by Karkoula et al. [38,39] regarding the artificial formation of some secoiridoid derivatives (mainly due to their reactivity with methanol (and water)), this topic is of a great interest. Our group has already discussed it in another publication [40], where we corroborated Karkoula´s finding, after carrying out the extraction of selected olive oil samples both with the very widely used methanol/water (60:40, *v*/*v*) mixture and acetonitrile. When acetonitrile replaces the methanol-water mixture as extractant, only 3 isomers of Ol Agl (at relatively low proportions) can be observed. The same can be said for Lig Agl (observing just one isomer (instead of 3)). As a consequence, it is possible to claim that as long as methanol (and probably water and/or their mixtures) is involved in the sample preparation (or has any interaction at any point of the analytical procedure with these compounds), the “artificial isomers” will show up. We also corroborated that the generation of artificial peaks (related to DOA and D-Lig Agl) in the chromatograms is not as serious as for Ol Agl and Lig Agl and could be even ignored (from a quantitative point of view).

Bearing in mind all the outcomes just mentioned and the fact that methanol-water mixtures have been extensively used (and are recommended by the IOC method) and are probably present (water for sure) in most of the LC mobile phases combinations employed to analyze phenols from VOOs, we decided to keep using the extraction protocol implying the use of the methanolic mix (whose repeatability had been exhaustively checked (relative standard deviations (RSD) < 7.12%)).

To conclude the argumentation, it is necessary to add that, from our point of view, ignoring the “artificial isomers” means underestimating their initial “native amount”, since they are formed from the native secoiridoids present in the VOO sample. Some other authors agree regarding this last point (Karkoulas’ team, among others). For further information, have a look at Bajoub et al. [40].

### 2.2. Quantification of Phenolic Compounds and Statistical Analysis: Effect of Cultivar and Crop Season

**Figure 2:**
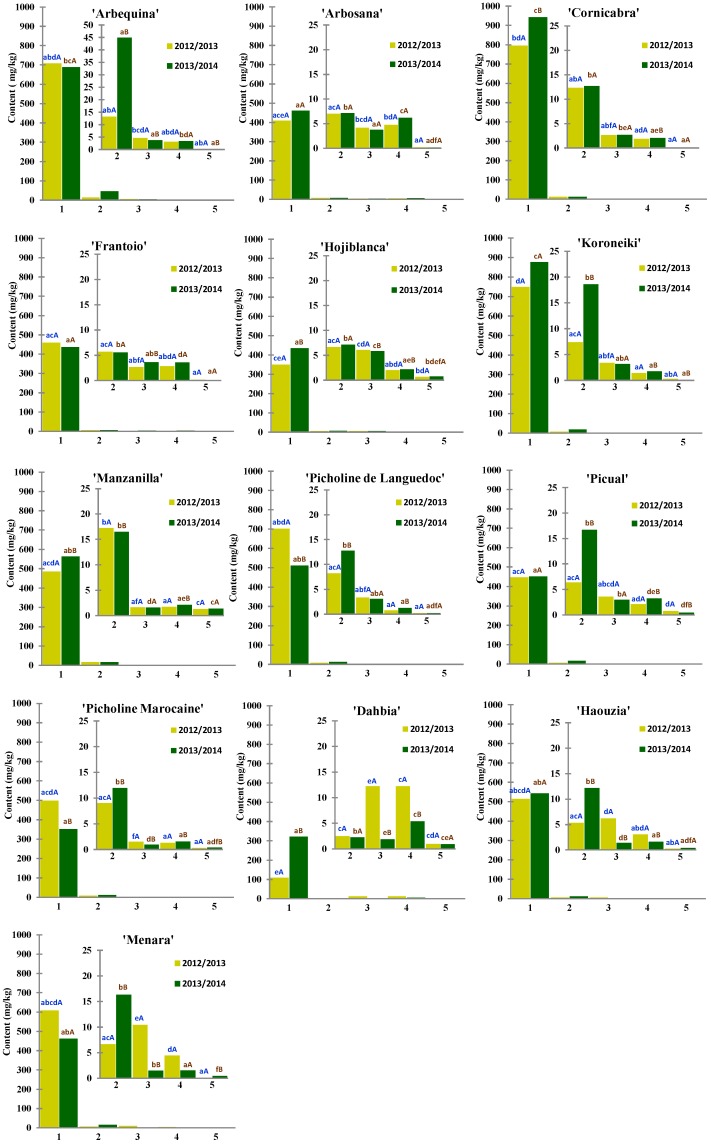
Total content of secoiridoids (**1**), simple phenols (**2**), flavonoids (**3**), lignans (**4**) and phenolic acids (**5**) of the studied monovarietal VOO samples, expressed in mg/kg. Significant differences are indicated with different lowercase letters (comparison among the 13 cultivars investigated in this study at the same crop season, *p* < 0.05) and with different capital letters (comparison between crop seasons for the same cultivar, *p* < 0.05).

In this work, total content of each chemical family (Figure 2) (calculated as the sum of the individual contents of the phenolic compounds belonging to each chemical class) and individual contents of the 33 identified compounds (Table 1, Table 2, Table 3 and Table 4) were determined by liquid chromatography electrospray ionization ion trap mass spectrometry (LC-ESI-IT MS).

As expected and can be deduced from Figure 2 and Table 1, Table 2, Table 3 and Table 4, in both cases (total content of each chemical family and individual phenolic compounds content), huge differences were observed among the studied oils due to the cultivar factor. These findings are in good agreement with previous works that highlighted the significant effect of the variety of origin on VOO phenolic fraction [34,37]. Furthermore, environmental factors, depicted by climatology of each season, exhibited a significant influence on the phenolic composition (regarding both total and individual contents) on almost all the investigated cultivars (Figure 2 and Table 1, Table 2, Table 3 and Table 4). Indeed, as can be observed from Table 1, the two seasons considered in this study greatly differed concerning the climatic conditions; important differences in total precipitations and average temperatures (minimum and maximum) per month were observed, especially in the months of olive fruit growing and before olive harvesting. This could result on a great inter-annual variability of the quantitative phenolic composition of the studied oils. In this respect, the effect of crop season climatology on the phenolic composition of VOO has been already established [32].

“Cornicabra” oils presented the highest total secoiridoids content in both crop seasons (mean content was 795.18 mg/kg in 2012/2013 and 942.64 mg/kg in 2013/2014), whereas the lowest mean concentration was calculated for “Dahbia” samples (108.59 and 321.06 mg/kg in crop seasons 2012/2013 and 2013/2014, respectively). Besides, in terms of total abundance, the second class of phenols was the group of simple phenols. That was observed for most of the cultivars under study, except for the Moroccan autochthonous cultivars “Haouzia” and “Menara”, which showed higher level of flavonoids than simple phenols’ content during the first crop season (over the second crop season, this category was the second most abundant phenolic class also for the oils from these cultivars). The same was observed for “Dahbia” variety, for which lignans were the second most abundant phenolic class in both crop seasons. Considering the simple phenols, “Manzanilla” (17.25 mg/kg) and “Arbequina” (44.91 mg/kg) were the cultivars with the highest mean contents over the first and second crop seasons, respectively, whereas the lowest values were again found for “Dahbia” oils in both crop seasons (2.51 mg/kg in 2012/2013 and 2.29 mg/kg in 2013/2014, respectively). As far as the total content on flavonoids is concerned, “Dahbia” and “Hojiblanca” showed the highest mean values, 12.20 mg/kg (1st season) and 5.87 mg/kg (2nd season), whilst the lowest values, in both crop seasons, were found for “Picholine Marocaine” oil samples (1.50 and 0.95 mg/kg in 2012/2013 and 2013/2014, respectively). With regard to the lignans, over the first crop season, the mean total content of the studied oils ranged between 12.24 and 0.78 mg/kg, in oils from “Dahbia” and “Picholine de Languedoc”, respectively; whereas during the second crop season mean values oscillated between 6.17 mg/kg (“Arbosana”) and 1.28 mg/kg (“Picholine de Languedoc”). Finally, the total content of phenolic acids (*p*-coum) was found between 1.28 mg/kg (in 2012/2013) and 1.40 mg/kg (in 2013/2014) in “Manzanilla” oil samples, whilst “Cornicabra” samples showed the lowest levels (0.07 mg/kg, in both crop seasons).

Different concentrations were logically found (for each phenolic compound) in each of the analyzed cultivars, finding significant differences among them. Moreover, as occurred for the total content for each chemical phenolic class, in almost all the investigated cultivars, the effect of crop season was statistically significant (*p* < 0.05). Table 1 illustrates these variations, showing the mean concentrations (±standard deviation) of the individual phenolic compounds determined by our LC-MS method.

Over the period considered by the current study, the phenolic fractions of the studied oils were found to mainly contain secoiridoids, regardless the cultivar of origin. Numerous reports have previously claimed this fact [29,33,34].

Concerning the secoiridoids group, results from Table 2, Table 3 and Table 4 show that the predominant compound in the analyzed oils mainly depend on the cultivar of origin and, in some cases, on the crop season. In this respect, over the studied period, DOA was by far the most abundant secoiridoid in oils from “Arbequina”, “Cornicabra”, “Hojiblanca”, “Koroneiki”, “Manzanilla”, “Picholine Marocaine” and “Picual” cultivars (mean concentrations ranged between 39.58 mg/kg, observed for samples from “Picholine Marocaine” cultivar (during the second crop season) and 350.41 mg/kg, found in “Arbequina” oils (first crop season), whereas in oils from “Arbosana” and “Frantoio”, D-Lig Agl showed the highest mean concentration values (between 80.45 and 135.98 mg/kg found, respectively, in “Frantoio” and “Arbosana” oils, both during the first crop season). For the other investigated cultivars, the most abundant compounds were different depending on the crop season. Indeed, over the first crop season, within the secoiridoids group, D-Lig Agl was the most abundant compound in oils from “Picholine de Languedoc” variety (156.39 mg/kg); Lig Agl showed the highest levels in oils from “Haouzia” (95.84 mg/kg) and “Menara” (113.01 mg/kg) cultivars; and Dehydro Ol Agl was the most abundant in oils from “Dahbia” variety (15.75 mg/kg). In contrast, during the second crop season, DOA seemed to be the most abundant secoiridoid in oils from “Picholine de Languedoc” (81.55 mg/kg), “Haouzia” (87.24 mg/kg) and “Menara” (70.68 mg/kg), while “Dahbia’ oils showed D-Lig Agl as their most prevalent individual complex phenol (93.73 mg/kg). Apart from the mentioned compounds, as detailed in Table 2, Table 3 and Table 4, the other identified secoiridoids were found in a wide range of concentrations in almost all the investigated cultivars (as depicted by the high standard deviation values obtained for each cultivar during the same crop season). Great differences between the two crop seasons were also observed.

As far as the simple phenols are concerned (Table 1), “Arbequina” oils were clearly outstanding in terms of their richness on Hyt-Ac (the most abundant simple phenol in those samples, with mean concentrations ranging between 10.84 and 26.63 mg/kg, observed for 1st and 2nd crop seasons, respectively). In the other studied cultivars, Ty was the most abundant simple phenol for almost all the varieties (except for “Koroneiki”), with amounts ranging from 1.73 mg/kg in oils from “Dahbia” (for the second crop season) to 11.74 mg/kg in olive oils from “Manzanilla” cultivar (for the first crop season). Hyt was found at concentration levels between 0.23 and 8.58 mg/kg, observed, respectively, in oils from “Dahbia” and “Koroneiki”, for the second crop season. O-Hyt was found at very low levels in the studied samples (mean concentration ranged from 0.11 and 1.03 mg/kg in oils from “Dahbia” and “Arbosana”, respectively, for the 2nd crop season).

When individual lignans are studied in depth, the evaluated cultivars could be divided in two groups, depending on their most abundant compound belonging to this chemical class. Thus, a first group including “Arbequina”, “Arbosana”, “Koroneiki”, ”Manzanilla”, “Picholine de Languedoc”, “Picual” and “Dahbia”, displayed higher levels of Pin than the other detected lignans (particularly, than Ac-Pin). Mean concentration values of this compound in oils from these varieties were between 0.50 and 6.41 mg/kg, found in oils from “Picholine de Languedoc” and “Dahbia” cultivars (first crop season), respectively (Table 1). The second group, which includes oils from “Cornicabra”, “Frantoio”, “Hojiblanca”, “Haouzia”, “Menara” and “Picholine Marocaine” was characterized by the predominance of Ac-Pin in the lignans’ group, with amounts ranging from 0.61 mg/kg in oils from “Picholine Marocaine” to 3.92 mg/kg in “Menara” samples (both observed for the first crop season). Finally, the Syr content of the studied oils was, in general, quite low in all the studied oils, regardless of the cultivar of origin; the obtained values were between 0.13 and 0.71 mg/kg and were found in oils from “Haouzia” (first crop season) and “Arbosana” (second crop season), respectively.

Moreover, the analysis of the individual flavonoids content showed that, except for “Dahbia” oils, Lut was the most abundant compound belonging to this family in oils from the other varieties, with levels ranging between 0.94 mg/kg (“Picholine Marocaine”, second crop season) and 5.54 mg/kg (“Menara”, first crop season). In the case of “Dahbia” oils, most of the samples from this variety seemed to contain more Apig than Lut. The mean Apig content in these oils was found between 1.82 mg/kg in 2012/2013 and 8.07 mg/kg in 2013/2014.

*p*-coum acid was found at relatively low levels in the studied oils, not exceeding the value of 1.4 mg/kg (in terms of mean amount). “Manzanilla” oils showed the highest level for the second crop season, whereas the lowest amount (0.07 mg/kg) was found in “Cornicabra” oils for both crop seasons.

In this study, the content of QA, was also determined (Table 1). The amount of this compound was within the range from 0.08 mg/kg found in “Dahbia” oils to 4.98 mg/kg found in ”Arbosana” samples, levels measured during the first and second seasons, respectively.

The comparison of the phenolic content values obtained in this study with those from other studies (investigating the phenolic composition of some of the considered Mediterranean varieties but in their region or country of origin), is quite difficult and some considerations have to be taken into account: the analytical methodologies can differ and, sometimes, the determined compounds are even quantified in terms of another commercially available pure standard; moreover, several characteristics of the analyzed samples can be very influential, particularly, the maturation stage of the extracted olive fruits, the oil extraction system used, the pedoclimatic conditions of a particular season/location, and the filtration and conservation conditions.

If our results are compared, just for illustrative purposes, with those obtained in previously published works by Bakhouche et al. [33], García-Villalba et al. [37] and Lozano-Sánchez et al. [34], where the authors used LC-MS approaches for the characterization of the phenolic profile of oils from “Arbequina”, “Hojiblanca”, “Picual” and “Manzanilla”, it can be seen that, in general, the qualitative profiles were very similar, as well as the concentration levels of some of the determined compounds.

In light of this detailed analysis of the phenolic composition of the studied oils, general traits or features of “Picholine Marocaine” oils (the most extended variety in Morocco) could be described, trying to compare the oils coming from this cultivar with Mediterranean cultivars introduced in Morocco, and with the three other autochthonous Moroccan varieties studied herein. Based on the results reported in Figure 2 and Table 1, Table 2, Table 3 and Table 4, it can be concluded that oils from “Picholine Marocaine” variety (in pedoclimatic conditions of Moroccan Meknès region) showed an "intermediate phenolic composition" as compared to the one found in both Mediterranean cultivars introduced to Morocco and the three autochthonous Moroccan varieties. Just one peculiar characteristic could be revealed for “Picholine Marocaine” oils: their low content on flavonoids. If we focus on finding differences (or peculiar characteristics) among the three autochthonous Moroccan varieties obtained through clonal selection from “Picholine marocaine” cultivar, it can be noticed that the phenolic composition of “Menara” and “Haouzia” oils were quite similar in general terms, and “Dahbia” oils showed very distinctive phenolic composition (as previously detailed in this paper) for each one of the characterized chemical classes.

Despite these findings, examination of the results obtained applying a *post-hoc* test (Tukey’s) to the phenolic profiles of the studied oils, we found that there was not any compound that could correctly classify the studied samples according to their varietal origin, since the mean values for each variable were not significantly different for all the 13 cultivars at the same time (Figure 2 and Table 1, Table 2, Table 3 and Table 4). The quantitative compositions of the analyzed oils also showed large differences in phenolic content within the same varietal origin and no characteristic concentration ranges could be set. That is why we decided to go for the application of chemometric data analysis to test the ability of the identified phenolic compounds for tracing the varietal origin of the oils under evaluation.

### 2.3. Chemometrics

#### 2.3.1. Principal Components Analysis

**Figure 3:**
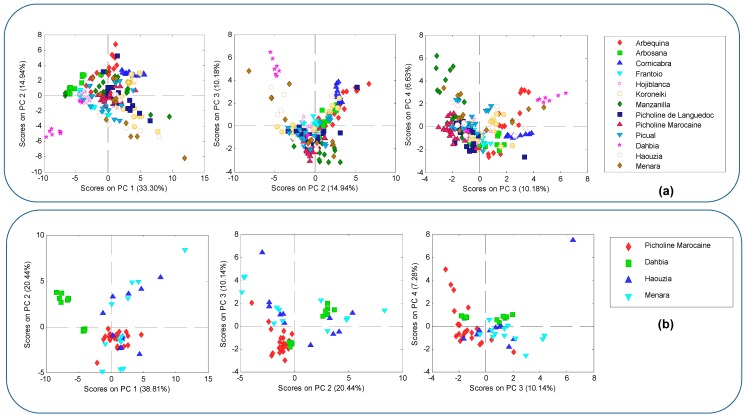
(**a**) Scores of samples from “Picholine Marocaine” and Mediterranean cultivars on the map defined by the first four principal components; and (**b**) Scores of samples from “Picholine Marocaine” and the three other investigated Moroccan cultivars on the map defined by the first four principal components.

An exploratory approach through non-supervised PCA, using as chemical descriptors the auto-scaled data of the phenolic content found in the analyzed olive oil samples, was performed to provide a data structure study over a reduced dimension, covering the maximum amount of the information present in the basic data, which allows to investigate any possible clustering of the samples on the basis of their varietal origin. Thus, in a first step, PCA was performed on the entire data set (including the samples from the 13 cultivars, the content of the 33 determined compounds, and the total content of the five phenolic chemical classes as dependent variables). From this analysis, 20 principal components (PCs) were identified; 9 of them were the most important (eigenvalues > 1). The first four components explained 65.06% of the total variability. The obtained data structure is shown in the graphic of scores, which were based on PC1 vs. PC2, PC2 vs. PC3, and PC3 vs. PC4 (Figure 3a). Examining the score plot of the objects in these sub-spaces, it was evident that no separation of the 13 cultivars was achieved, except for oils from “Dahbia” variety (in particular, for one of the seasons), which were quite differentiated from those of other cultivars. Afterwards, to check possible dissimilarities among samples from Moroccan varieties, a second PCA was then applied just to data from the four Moroccan cultivars investigated in this study. Considering the eigenvalues > 1, seven PCs contained 89.89% of total variance. Figure 3b provides an overview of the natural clustering of the studied Moroccan olive oils samples on PC1 vs. PC2, PC2 vs. PC3, and PC3 vs. PC4 sub-spaces. As can be observed in this Figure, the samples from “Dahbia” variety (from one season) were again the only ones showing a clear clustering and were distinct from those of the other three considered groups, which were strongly overlapped. This may be explained considering the fact that the phenolic composition of olive oils from “Haouzia” and “Menara” is, to some extent, close or similar to their parental cultivar “Picholine Marocaine”. In other words, although differences between these cultivars can be found (Figure 2 and Table 1, Table 2, Table 3 and Table 4), they are not distinct enough to allow the separation of samples from these varieties based only on non-supervised chemometric methods. Hence, the importance of employing supervised chemometric techniques with the purpose of obtaining classification rules for assigning categories to samples.

#### 2.3.2. Linear Discriminant Analysis Models

As stated above, with the aim of testing the capability of phenolic compounds variables to distinguish “Picholine Marocaine” olive oils from the other studied oils, two LDA models were constructed: first of all, LDA was applied to a matrix containing 168 objects (which correspond to the samples from “Picholine Marocaine” and the nine Mediterranean cultivars); secondly, LDA was carried out on a matrix composed by using samples from “Picholine Marocaine” and the three autochthonous Moroccan varieties (59 samples). Another LDA model was also computed with the aim of discriminating among oils obtained from the nine Mediterranean cultivars and the three Moroccan varieties “Dahbia”, “Haouzia” and “Menara”. In the three LDA models constructed in this study, 38 predictors (the same as in PCA treatment) were used and no variable reduction was applied.

The magnitude of Wilks’ Lambda, which reflects the proportion of the variance in the dataset that is not accounted for the model, was evaluated and significant values were obtained for the three LDA models built. Likewise, the uniformity of variability was tested (Box M index) and results were insignificant at the 95% confidence level, showing the existence of uniformity of sample variability for each varietal origin considered in the three LDA models. Moreover, the stepwise algorithm was applied to extract the best discriminant variable/s separating the studied samples according to their varietal origin. This discriminant procedure enters or removes variables by analyzing their effects on the discrimination of the groups based on the Wilks’ lambda criterion. The selected variables for each model were the following: (a) Ac-Pin, Apig, Dehydro Ol Agl, DOA, EA, Hy D-Ol Agl, Hy-EA, Hyt, Hyt-Ac, Lig Agl IS1, Methyl Ol Agl, Ol Agl IS2, Ol Agl IS5, Ol Agl IS6, *p*-coum, Pin, total flavonoids, total lignans, total secoiridoids and total simple phenols for the first LDA model; (b) 10 Hy-Ol Agl, Apig, D-Ald-D EA, Dehydro Ol Agl, D-Lig Agl, EA, Methyl D-Ol Agl, Methyl Ol Agl, Ol Agl, Ol Agl IS4, Ol Agl IS6, *p*-coum, Pin, total flavonoids and Ty for the second LDA model; and (c) 10 Hy-Ol Agl, Ac-Pin, Apig, D-Ald-D EA, Dehydro Ol Agl, Desoxy-EA, D-Lig Agl, DOA, EA, H-D-Ol Agl, Hy-EA, Hyt, Hyt-Ac, Lig Agl, Lig Agl IS1, Lig Agl IS2, Lig Agl IS3, Lut, Methyl D-Ol Agl, Methyl Ol Agl, O-Hyt, Ol Agl, Ol Agl IS1, Ol Agl IS2, Ol Agl IS3, Ol Agl IS4, Ol Agl IS5, Ol Agl IS6, *p*-coum, Pin, QA, Syr, total flavonoids, total lignans, total secoiridoids, total simple phenols and Ty for the third LDA model. Besides, to evaluate the prediction power of the obtained models, “leave-one-out” cross-validation was applied. Tables S1–S3 show recognition and prediction abilities achieved by each LDA model.

Thus, as can be seen from the Table S1, the first LDA model, constructed with the phenolic compounds contents of “Picholine Marocaine” and the other studied Mediterranean varieties, was able to correctly discriminate “Picholine Marocaine” samples from the other monovarietal VOOs. 100% of correct classification and prediction were obtained for samples from this Moroccan variety. Similar results (100% in both classification and cross-validation) were obtained for other three monovarietal VOOs group: “Cornicabra”, “Hojiblanca” and “Manzanilla”. Very satisfactory results were also obtained for “Arbequina” and “Koroneiki” VOOs, which were 100% correctly classified, but the percentages obtained after cross-validation slightly decreased to 87.50% and 94.44%, respectively. Moreover, interesting results were obtained for “Frantoio” samples (93.75% in both classification and cross-validation), “Picual” (88.89% achieved in both classification and cross-validation) and “Arbosana” (86.67 in both classification and cross-validation). The worst classification and prediction abilities were observed for “Picholine de Languedoc” samples, where only 85.00% and 80.00% of them were correctly classified and predicted, respectively.

The overall accuracy of the LDA model was, in any case, very satisfactory, with values of 95.24% in classification and 92.86% in prediction.

The LDA classification results of samples from the four Moroccan groups are shown in the Table S2. The discriminant functions achieved a classification ability of 100%, whereas their overall predictive ability was of about 86.21% (explained by the eight samples which were not correctly predicted).

An analysis of these mispredicted samples showed that 4 (of 12) “Menara” samples were predicted as “Haouzia” oils and one as “Picholine Marocaine” sample; two “Haouzia” samples were predicted as “Menara” and “Picholine Marocaine” oils, and one “Picholine Marocaine” sample fell within the “Menara” group. Only “Dahbia” samples were 100% correctly predicted, fact which again confirms the distinctive phenolic composition of the oils from this cultivar (comparatively to the other Moroccan varieties). In general, the results obtained herein (Table S2) indicate satisfactory performances of the LDA model constructed for the classification (100%) and prediction (95.83%) of monovarietal “Picholine Marocaine” olive oils among the other autochthonous Moroccan cultivars considered by this study.

Furthermore, we tried to assess the potential of the phenolic profiles for the discrimination between Mediterranean olive oil samples and those obtained from the three Moroccan cultivars “Dahbia”, “Haouzia” and “Menara”. The obtained results (Table S3) show that the LDA model had overall classification and prediction abilities of 93.26% and 84.83%, respectively. When focusing on the obtained results for each monovarietal olive oil class, it can be observed that the built LDA model gave excellent results with a success rate of 100% in both classification and cross-validation for three monovarietal groups “Cornicabra”, “Dahbia” and “Hojiblanca”. Classification and prediction were also reasonably successful for other cultivars, such as “Koroneiki” (94.44% in both classification and cross-validation), “Arbequina” (93.75% in both classification and cross-validation) and “Frantoio” (93.75% for classification and 87.50% for cross-validation). In contrast, the worst results in classification (81.82%) and prediction (50.00%) were found for Moroccan samples from “Haouzia” and “Menara” varieties, respectively. Indeed, as shown by the results reported on Table S3, the low rate obtained in classification for “Haouzia” samples can be related to the misclassification of 2 of the 11 samples from this variety as “Cornicabra” and “Menara” oils, whereas only 6 from the 12 Menara samples were well predicted. The other 6 were mispredicted as “Frantoio” (one sample), “Picholine de Languedoc” (one sample) and “Haouzia” (4 samples) oils. The latter also showed low rate of correct prediction (54.55%), being among the 5 misspredicted samples from this class 1 “Cornicabra”, 1 “Manzanilla” and 3 classified as “Menara”. This proves that oil samples from these two Moroccan varieties (obtained by clonal selection from “Picholine Marocaine” cultivar) exhibit a quite similar phenolic composition, which makes their discrimination difficult.

## 3. Materials and Methods

### 3.1. Olive Samples Harvest, Oil Extraction and Physico-Chemical Quality Evaluation

Olive fruits sampling was performed over two consecutive crop seasons (2012/2013 and 2013/2014) on randomly selected trees, representing the 13 olive cultivars listed in Table 5, all grown in the experimental olive grove of the Agro-pôle Olivier National School of Agriculture of Meknès, Morocco. Pest control, pruning, irrigation and fertilization practices were done following current olive orchards management practices. This location belongs to the Mediterranean bioclimate. Information about trees plantation date and spacing, as well as experimental olive grove soil characteristics and climatic conditions during the two crop seasons considered in this study, are summarized in Table 5.

From each one of the 13 cultivars investigated in this study, good quality, fresh and healthy fruits samples (30 kg each one) were randomly hand-picked from the selected trees, starting from middle of October till the end of December, collected in ventilated plastic crates and immediately sent to the laboratory for oil extraction. In the laboratory, before oil extraction, each sample was properly homogenized and 100 fruits were randomly sampled to carry out the determination of the ripening index, following the method proposed by the Agronomic Station of Jaén (Spain), based on the evaluation of the olive skin and pulp colors [41]. Thus, to avoid possible influence of the fruits maturity stage on the phenolic profiles of the studied oils, in this study, only samples picked at a ripening index within the range 3.0–3.5 were considered. This range is commonly advised for the production of high quality olive oils in Meknès region [42]. A total of 203 samples were considered, the remaining were kept for other experiments. Information about the number of samples per cultivar and crop-season are reported in Table 5.

Afterwards, oil was extracted using an Oliomio laboratory mill (Oliomio, Italy) simulating two-phase commercial oil-extraction system. The operating mode of this instrument has been described in detail by Bajoub et al. [32]. Briefly, olives were washed, deleafed, and crushed and the resulting paste was mixed at 28–30 °C for 45 min, and decanted at 23–27 °C without the addition of any water. Obtained oil was filtered to remove impurities, transferred into dark glass bottles without headspace and immediately frozen and stored at −18 °C for further analysis.

To evaluate the physico-chemical quality of the obtained oils, regulated criteria (free fatty acids content (given as percentage of oleic acid (%)), peroxide value (expressed as milliequivalents of active oxygen per kilogram of olive oil (meq O_2_/kg)) and *K*_232_ and *K*_270_ extinction coefficients (calculated from absorption at 232 and 270 nm, respectively) were determined, in triplicate, for each studied oil sample by using the analytical methodologies described in the European Union Standard Methods Regulations 2568/91 and the subsequent amendments [43]. Obtained results (Table 5) allow classifying all the studied oils within the “extra virgin” category.

### 3.2. Application of a LC-MS Analytical Methodology to Establish the Phenolic Composition of the Studied Oils

#### 3.2.1. Chemicals and Reagents

All solvent used in this study were of analytical or LC-MS grade purity (depending on if they were used for the extraction or chromatographic analysis) and used without further purification. Acetonitrile and acetic acid were purchased from Lab-Scan (Dublin, Ireland) and Panreac (Barcelona, Spain), respectively. Methanol and *n*-hexane were obtained from Panreac (Barcelona, Spain). Double-deionized water with conductivity of 18.2 MΩ was purified with a Milli-Q-system (Millipore, Bedford, MA, USA). Standards of 3,4-dihydroxyphenylacetic acid (DOPAC), Hyt, Ty, Lut, Apig, *p*-coum, and QA were purchased from Sigma-Aldrich (St. Louis, MO, USA) and Pin was acquired from Arbo Nova (Turku, Finland). Oleuropein was purchased from Extrasynthese (Lyon, France).

#### 3.2.2. Preparation of Standards and Quality Control (QC) Samples

Stock solution at 500 mg/L of each one of the above-mentioned standards, was prepared in methanol. Subsequently, to prepare working standard solutions, the appropriate volumes of the stock solution, with the exception of DOPAC (which was used as internal standard (ISt)), were took and diluted serially in methanol to yield final concentrations of 0.5, 1, 2.5, 5, 12.5, 25, 35, 50, 100, 150 and 250 mg/L. QC samples at a concentration of 5 mg/L were prepared in the same way as the calibration standards, and were used to check the stability of the system over the different sequences carried out. Analytical standards solutions and QC samples were stored at −20 °C.

#### 3.2.3. Liquid-Liquid Extraction

Phenolic compounds were extracted in triplicate from each sample as follows: an aliquot of 2.00 g of oil was accurately weighted into a 12 mL glass centrifuge tube with a screw cap. A volume of 25 μL of the ISt solution were added, methanol was evaporated using nitrogen, and the phenolic compounds were extracted after adding 1 mL of *n*-hexane by using 2 mL of methanol/water (60:40, *v*/*v*). The mixture was firstly vortexed for 1 min and then centrifuged at 3500 rpm for 6 min at room temperature. The methanol/water fraction was collected, the residue was re-extracted twice under the same conditions for recovering the totality of phenolic compounds, and the three fractions were combined. Then, the solvent was removed, using a rotary evaporator under vacuum at 30 °C and reduced pressure. Subsequently, the obtained dry extract was reconstituted in 1 mL of methanol. The final extracts were filtered through 0.22 μm membrane (nylon) syringe filters and stored at −18 °C until analyzed.

#### 3.2.4. LC-MS Analysis

The phenolic extracts obtained were, in a first step, analyzed using a LC coupled to a microTOF-Q IITM mass spectrometer (Bruker Daltonik, Bremen, Germany) by an electrospray ionization source. The LC system was an Agilent 1260 LC system (Agilent Technologies, Waldbronn, Germany). The aim of this first step was to achieve the qualitative characterization of the phenolic fraction of representative samples of each one of the studied varieties. Afterwards, another LC-MS platform was used, coupling the same LC system to a Bruker Daltonics Esquire 2000™ Ion Trap (Bruker Daltonik, Bremen, Germany) for quantitative purposes. In both cases, the mass spectrometers were operating in negative ionization mode. LC-MS analyses were carried out by applying a previously optimized and validated analytical method [32]. Thus, chromatographic separation was carried out in a Zorbax C18 analytical column (4.6 × 150 mm, 1.8 μm particle size; Agilent Technologies, Waldbronn, Germany) protected by a guard cartridge of the same packing, operating at room temperature was used. The selected flow rate was 0.8 mL/min, and a volume of 10 μL was injected for all the methanolic extracts obtained from the samples. The mobile phase was composed of solvent A (water with 0.5% acetic acid) and solvent B (acetonitrile). Gradient elution was programmed as follows: 0 to 10 min, 5%–30% B; 10 to 12 min, 30%–33% B; 12 to 17 min, 33%–38% B; 17 to 20 min, 38%–50% B; 20 to 23 min, 50%–95% B. Finally, the B content was decreased to the initial conditions (5%) in 2 min. The total run time was 25 min, and the column equilibration time between each run was 2.5 min.

For the mass spectrometry analysis, the following parameters were adopted for IT mass spectrometer (and similar ones for microTOF-Q II ^TM^): capillary voltage, 3200 V; drying gas (N_2_) flow and temperature, 9 L/min and 300 °C, respectively; nebulizer pressure, 30 psi; scan range, 50–800 *m*/*z*. In IT MS, Ion Charge Control (ICC) was set at 10,000. MS data were processed through the software DataAnalysis 4.0 (Bruker Daltonik).

The identification and assignation of phenolic compounds was performed by comparing their retention times and accurate *m*/*z* values obtained by MS and MS^2^ with the mass spectra from corresponding pure standards tested under the same conditions and with previously published results from our research group or some other groups working in this field. For the accurate mass data (TOF MS), the software DataAnalysis 4.0 provided a list of possible elemental formulas by using the SmartFormula^TM^ Editor tool, which gives and rates plausible molecular formulas consistent with the accurate mass measurement and the true isotopic pattern (using the masses and intensities of each isotope for doing a comparison of the theoretical with the measured isotope pattern (mSigma value)).

For quantitative purposes, triplicate injections were made for each standard solution and sample; and the calibration curves obtained by LC-IT MS analysis were used for quantification of all the identified analytes. Linearity ranges for calibration curves were specified and the limits of detection (LOD) and quantification (LOQ) were determined. More detail about method validation and phenolic compounds quantification can be seen in Bajoub et al. [32].

For each studied variety, the quantitative results (Table 1, Table 2, Table 3 and Table 4) for the identified phenolic compounds were expressed as mean ± standard deviations (mean ± SD) considering results of all the samples from the same variety (and not just the SD coming from the three different replicate of the same sample). In other words, calculated means (arithmetic means) refer to the central value of quantitative dataset obtained considering all the analyzed samples from the same variety, while the SD describes the dispersion of this dataset from its mean (intra-variety variability). These mean and SD results should not be considered to get an idea about the repeatability of the analytical methods used. The total content for each chemical category is also given and was calculated as the sum of the individual phenols belonging to that chemical class.

It should be highlighted that in order to simplify data visualization for readers, quantitative results were divided into four parts (Table 1, Table 2, Table 3 and Table 4). The results of one-way ANOVA were included in each table, evaluating differences among all the investigated cultivars, as well as the influence of the crop season on their phenolic content.

### 3.3. Statistical Analysis

Significant differences in phenolic compounds composition (*p* < 0.05) among varieties and crop seasons were assessed with one-way analysis of variance (ANOVA) followed by *post-hoc* Tukey’s test. SPSS statistical package software (SPSS for Windows, Version 20, SPSS Inc., and Chicago, IL, USA) was used for the statistical analysis of the data. For varietal classification of the studied oils, the obtained quantitative data were subjected to PCA and LDA using XLSTAT software version 2015.04.1 (Addinsoft, Paris, France).

## 4. Conclusions

In conclusion, the data presented in this study are the first published on the phenolic composition of the studied cultivars in the Moroccan pedoclimatic conditions (not considering “Picholine Marocaine” *cv*.). The determination of the phenolic composition of the studied monovarietal olive oils could be of great importance to select cultivars with oils showing the highest nutritional value and promoting them amongst consumers. Besides, the considerable differences among the studied cultivars in terms of the content of phenolic compounds confirm that the genotype exhibits a major influence on the phenolic fraction of olive oil. Likewise, the effect of the crop season climatology was very significant. Finally, the application of LDA made possible the correct discrimination of “Picholine Marocaine” olive oils, as the main one produced in Morocco, from the other studied monovarietal olive oils categories. These results can strengthen authentication controls of Moroccan VOOs certified as monovarietal from this cultivar.

## Figures and Tables

**Table 1:** Mean ± standard deviation (mg/kg) of some of the phenolic compounds determined in the studied monovarietal olive oils.

Variety	QA	O-Hyt	Hyt	Ty	*p*-Coum	Hyt-Ac	Syr	Lut	Apig	Pin	Ac-Pin
	**2012/2013**										
**Arbequina**	0.46 abA ± 0.19	0.14 aA ± 0.02	0.47 aA ± 0.09	1.75 aA ± 0.54	0.31 abA ± 0.15	10.84 aA ± 6.47	0.22 aA ± 0.07	4.27 abcfA ± 0.89	0.35 aA ± 0.09	1.51 aA ± 0.33	1.39 aA ± 0.35
**Arbosana**	0.59 abA ± 0.58	0.19 aA ± 0.12	1.71 abA ± 2.62	2.91 acA ± 2.29	0.18 a ± 0.06	2.19 bA ± 3.93	0.69 bA ± 0.34	3.08 acA ± 1.23	1.08 adA ± 0.61	2.31 abA ± 1.11	1.78 aA ± 0.78
**Cornicabra**	0.24 abA ± 0.04	0.33 abA ± 0.02	4.30 bcA ± 0.33	6.39 acA ± 0.43	0.07 a ± 0.01	1.32 bA ± 0.15	0.16 aA ± 0.01	2.54 acdeA ± 0.39	0.08 aA ± 0.01	0.61 adA ± 0.03	1.12 aA ± 0.06
**Frantoio**	0.52 aA ± 0.26	0.22 aA ± 0.09	0.81 aA ± 0.55	4.07 acA ± 2.54	0.13 a ± 0.09	0.59 bA ± 0.19	0.21 aA ± 0.04	2.45 cdeA ± 0.79	0.20 aA ± 0.09	0.74 adA ± 0.19	1.89 adA ± 0.73
**Hojiblanca**	0.44 abA ± 0.37	0.19 aA ± 0.06	0.55 aA ± 0.29	4.53 aA ± 1.75	0.67 b ± 0.28	1.35 bA ± 0.54	0.27 acA ± 0.08	5.27 abfA ± 1.01	0.80 adA ± 0.18	0.80 adA ± 0.21	0.95 aA ± 0.29
**Koroneiki**	0.45 abA ± 0.30	0.16 aA ± 0.07	1.61 aA ± 1.24	4.27 acA ± 1.79	0.29 ab ± 0.09	1.31 bA ± 1.19	0.17 aA ± 0.05	3.07 cA ± 0.69	0.29 aA ± 0.11	0.65 dA ± 0.14	0.62 aA ± 0.11
**Manzanilla**	0.43 aA ± 0.36	0.16 aA ± 0.05	4.05 cA ± 3.03	11.74 bA ± 5.98	1.28 c ± 0.47	1.30 bA ± 1.02	0.30 acA ± 0.21	1.57 deA ± 0.29	0.07 aA ± 0.05	0.74 adA ± 0.66	0.72 aA ± 0.64
**P-Languedoc**	4.89 bA ± 7.38	0.84 bA ± 1.04	1.31 aA ± 0.80	5.67 acA ± 3.08	0.24 a ± 0.16	0.36 bA ± 0.22	0.19 aA ± 0.06	3.12 acA ± 1.21	0.26 aA ± 0.13	0.50 dA ± 0.14	0.08 acA ± 0.15
**P-Marocaine**	1.74 abA ± 3.37	0.26 aA ± 0.33	1.23 aA ± 0.57	6.11 cA ± 0.99	0.29 a ± 0.09	1.43 bA ± 1.04	0.21 aA ± 0.06	1.47 deA ± 0.44	0.04 bA ± 0.07	0.46 dA ± 0.11	0.61 aA ± 0.15
**Picual**	0.28 aA ± 0.09	0.24 aA ± 0.16	1.47 aA ± 1.43	4.33 acA ± 1.01	0.75 bc ± 0.30	0.34 bA ± 0.22	0.22 aA ± 0.04	3.30 acA ± 1.04	0.28 aA ± 0.11	1.80 abA ± 0.72	0.13 acA ± 0.11
**Dahbia**	0.08 aA ± 0.03	0.19 aA ± 0.03	0.35 aA ± 0.13	1.88 aA ± 0.43	1.00 bc ± 0.11	0.09 bA ± 0.10	0.49 bcA ± 0.09	4.13 abcfA ± 0.50	8.07 cA ± 1.14	6.41 cA ± 0.71	5.33 bcA ± 1.19
**Haouzia**	1.18 abA ± 2.03	0.21 abA ± 0.19	0.51 aA ± 0.29	3.63 acA ± 1.90	0.29 ab ± 0.40	0.99 bA ± 0.80	0.13 aA ± 0.05	3.98 abcfA ± 1.74	2.22 dA ± 1.30	0.37 dA ± 0.11	2.56 cdA ± 0.92
**Menara**	0.91 abA ± 1.35	0.19 aA ± 0.08	1.02 aA ± 1.02	4.91 acA ± 2.31	0.14 a ± 0.09	0.54 bA ± 0.52	0.17 aA ± 0.09	5.54 bfA ± 1.03	4.86 eA ± 3.31	0.37 dA ± 0.19	3.92 bdA ± 4.66
	**2013/2014**										
**Arbequina**	0.39 aA ± 0.14	0.30 aB ± 0.06	6.38 bcdB ± 2.67	11.60 bB ± 3.28	0.11 bB ± 0.01	26.63 aB ± 32.66	0.36 aB ± 0.06	3.39 aB ± 0.21	0.32 bdgfA ± 0.03	1.34 aA ± 0.15	1.62 bA ± 0.54
**Arbosana**	4.98 aA ± 11.67	1.03 aA ± 1.73	1.30 aA ± 1.92	3.28 cedA ± 4.00	0.16 bfA ± 0.11	1.53 bA ± 1.25	0.78 bA ± 0.23	2.61 cA ± 0.45	1.18 cA ± 0.21	3.38 bB ± 0.45	2.00 bcA ± 0.54
**Cornicabra**	0.24 aA ± 0.01	0.35 aA ± 0.03	4.33 abdA ± 0.39	6.64 acedA ± 0.36	0.07 abA ± 0.01	1.39 bA ± 0.09	0.20 aB ± 0.01	2.55 acA ± 0.22	0.10 fagB ± 0.01	0.66 eB ± 0.03	1.21 abA ± 0.14
**Frantoio**	0.74 aA ± 0.15	0.45 aB ± 0.03	0.79 aA ± 0.36	4.08 cedA ± 0.62	0.11 bA ± 0.03	0.25 bB ± 0.16	0.32 aB ± 0.08	3.18 aB ± 0.29	0.42 dfgB ± 0.13	0.92 aeA ± 0.22	2.30 cA ± 0.67
**Hojiblanca**	0.36 aA ± 0.27	0.17 aA ± 0.03	0.51 aA ± 0.30	5.41 deA ± 2.14	0.76 cefA ± 0.33	1.05 bA ± 0.76	0.32 aA ± 0.09	5.09 bA ± 1.08	0.79 eA ± 0.18	0.85 aeA ± 0.18	1.03 abA ± 0.25
**Koroneiki**	1.68 aB ± 1.51	0.36 aB ± 0.08	8.58 bcB ± 2.63	7.45 acdB ± 2.35	0.10 abB ± 0.02	2.21 bB ± 0.28	0.25 aB ± 0.04	2.88 aA ± 0.29	0.28 fgA ± 0.04	0.94 aeB ± 0.12	0.56 adeA ± 0.09
**Manzanilla**	0.19 aA ± 0.03	0.13 aA ± 0.02	3.82 abdA ± 3.29	10.76 abA ± 5.55	1.40 dA ± 0.57	1.77 bA ± 1.52	0.34 aA ± 0.24	1.52 cdA ± 0.23	0.07 aA ± 0.06	0.91 aeA ± 0.92	0.86 adA ± 0.78
**P-Languedoc**	0.59 aA ± 0.31	0.34 aA ± 0.05	2.19 adB ± 0.84	9.48 abdB ± 1.22	0.24 abfA ± 0.05	0.75 bB ± 0.21	0.30 aB ± 0.09	2.85 aA ± 0.72	0.27 bgA ± 0.19	0.67 eB ± 0.16	0.32 deB ± 0.18
**P-Marocaine**	0.59 aA ± 0.38	0.27 aA ± 0.07	2.21 adA ± 1.69	8.53 abdB ± 1.56	0.36 abfB ± 0.06	0.95 bA ± 0.25	0.26 aB ± 0.04	0.94 cdB ± 0.17	0.01 aA ± 0.02	0.57 eB ± 0.11	0.71 adeA ± 0.09
**Picual**	0.80 aB ± 0.58	0.55 aB ± 0.09	4.78 dB ± 2.74	9.19 abdB ± 2.02	0.45 acfB ± 0.08	2.20 bB ± 1.97	0.31 aB ± 0.04	2.77 aA ± 0.27	0.19 abfgB ± 0.06	2.80 cB ± 0.20	0.13 eA ± 0.04
**Dahbia**	0.17 aB ± 0.01	0.11 aB ± 0.01	0.23 aA ± 0.03	1.73 deA ± 0.16	0.92 ceA ± 0.05	0.22 bB ± 0.04	0.32 aB ± 0.04	0.59 cdB ± 0.25	1.82 afgB ± 0.01	4.22 dB ± 0.08	0.60 adeB ± 0.02
**Haouzia**	0.42 aA ± 0.21	0.32 aA ± 0.05	3.68 abdB ± 1.40	7.61 abcdB ± 2.02	0.40 abfA ± 0.17	0.54 bA ± 0.30	0.31 aB ± 0.06	1.34 cB ± 0.38	0.05 aB ± 0.03	0.50 eB ± 0.04	0.80 adeB ± 0.18
**Menara**	0.41 aA ± 0.16	0.41 aB ± 0.11	4.07 abdB ± 2.72	11.41 abB ± 2.58	0.45 cfB ± 0.10	0.44 bA ± 0.24	0.32 aB ± 0.04	1.43 cdB ± 0.27	0.05 aB ± 0.03	0.57 eB ± 0.11	0.64 adeA ± 0.13

Significant differences in the same column are indicated with different lowercase letters (comparison among the 13 cultivars investigated in this study at the same crop season, *p* < 0.05) and with different capital letters (comparison between crop seasons for the same cultivar, *p* < 0.05). QA, Hyt, Ty, Pin, Lut, Apig, and *p*-coum were quantified in terms of their commercial pure standards. O-Hyt and Hyt-Ac were quantified in terms of Hyt. Lignans (Syr and Ac-Pin) were quantified in terms of Pin. P-Languedoc: Picholine de Languedoc; P-Marocaine: Picholine Marocaine.

**Table 2:** Mean ± standard deviation (mg/kg) of some of the phenolic compounds determined in the studied monovarietal olive oils.

Compound		Arbequina	Arbosana	Cornicabra	Frantoio	Hojiblanca	Koroneiki	Manzanilla	P-Languedoc	Picual
**D-Ald-D EA**	**2012/2013**	0.40 aA ± 0.27	2.24 aA ± 3.06	1.50 aA ± 0.22	1.47 aA ± 2.92	0.72 aA ± 0.43	0.75 aA ± 1.14	19.42 bA ± 18.75	2.59 aA ± 3.78	1.58 aA ± 1.45
**Desoxy-EA**		6.83 acA ± 3.54	5.79 ac ± 9.59	2.42 a ± 0.15	3.99 a ± 5.42	9.38 acd ± 13.76	56.30 b ± 23.04	27.08 cd ± 25.17	32.79 d ± 13.70	6.67 ac ± 11.17
**Hy-EA**		0.11 aA ± 0.02	0.10 a ± 0.04	1.51 b ± 0.16	0.20 a ± 0.10	0.20 a ± 0.05	0.18 a ± 0.09	0.16 a ± 0.07	0.24 a ± 0.07	0.19 a ± 0.06
**EA**		27.97 aA ± 10.54	25.09 aA ± 11.83	58.68 bcA ± 5.49	49.36 cA ± 9.05	26.21 aA ± 8.58	20.27 adA ± 8.94	26.88 aA ± 7.93	25.45 aA ± 8.17	28.29 aA ± 12.66
**Hy D-Ol Agl**		1.17 aA ± 0.59	0.41 abA ± 0.49	3.71 cA ± 0.14	0.34 bA ± 0.25	0.37 abA ± 0.21	0.81 abA ± 0.46	0.76 abA ± 0.70	0.71 abA ± 0.41	0.42 abA ± 0.57
**DOA**		350.41 aA ± 133.43	121.82 bA ± 162.41	205.65 acA ± 8.03	65.70 bA ± 35.16	87.37 bA ± 35.75	143.90 bA ± 60.32	93.94 bA ± 70.06	138.58 bA ± 39.23	71.25 bA ± 107.56
**10 Hy-Ol Agl**		0.19 aA ± 0.06	0.65 abA ± 1.37	3.48 bA ± 0.24	0.45 aA ± 0.11	0.27 aA ± 0.10	0.34 aA ± 0.13	0.29 aA ± 0.19	1.82 abA ± 4.98	0.31 aA ± 0.15
**Methyl D-Ol Agl**		2.22 aA ± 2.08	3.53 abA ± 5.33	11.30 bcdgA ± 1.06	14.89 dgA ± 1.41	5.98 abcA ± 3.95	16.59 dA ± 4.18	9.62 abgA ± 4.79	14.11 egA ± 6.82	13.85 degA ± 3.34
**D-Lig Agl**		203.04 aA ± 44.28	135.98 abcfA ± 36.20	190.46 abA ± 5.27	80.45 fA ± 34.30	75.32 efA ± 25.73	106.13 cfA ± 50.04	77.47 fA ± 32.01	156.39 abcA ± 50.19	55.89 dA ± 51.71
**Dehydro Ol Agl**		1.67 aA ± 1.64	1.13 aA ± 0.73	1.52 aA ± 0.23	5.56 bceA ± 1.71	2.97 aceA ± 0.32	1.29 aA ± 0.78	1.93 aA ± 1.01	1.85 aA ± 0.57	4.39 ceA ± 1.70
**Methyl Ol Agl**		1.80 abdA ± 0.64	1.44 aA ± 0.47	0.83 aA ± 0.05	2.86 bcdA ± 0.78	1.95 abdA ± 0.46	3.32 bcdA ± 0.87	1.33 adA ± 0.70	1.25 aA ± 0.48	1.76 abdA ± 0.64
**D-Ald-D EA**	**2013/2014**	2.74 aB ± 0.42	20.13 aA ± 48.34	1.46 aA ± 0.09	3.33 aA ± 4.77	0.49 aA ± 0.22	4.82 aB ± 2.32	10.59 aA ± 11.56	1.73 aA ± 1.21	14.45 aB ± 12.39
**Desoxy-EA**		5.65 aA ± 0.29	1.62 aA ± 0.62	2.81 abB ± 0.11	2.13 aA ± 1.57	8.95 abA ± 13.65	10.64 abB ± 1.77	25.82 bcA ± 32.55	45.44 bA ± 21.23	3.94 aA ± 2.04
**Hy-EA**		1.04 bB ± 0.24	0.60 ceB ± 0.38	1.60 dA ± 0.09	0.50 aceB ± 0.07	0.14 aA ± 0.05	0.42 aceB ± 0.11	0.15 aA ± 0.12	0.28 aceA ± 0.11	0.53 eB ± 0.19
**EA**		55.03 aA ± 35.29	60.33 bcA ± 42.65	61.30 abcA ± 5.95	89.04 bcB ± 20.11	38.52 abdA ± 15.46	28.60 abdB ± 3.03	22.42 adA ± 8.21	43.57 abdB ± 14.08	36.85 abdA ± 5.22
**Hy D-Ol Agl**		3.30 aB ± 0.48	1.10 bA ± 0.83	4.23 aB ± 0.28	0.39 bA ± 0.32	0.14 bB ± 0.07	2.15 cB ± 0.68	0.68 bA ± 0.75	0.46 bA ± 0.19	0.86 bA ± 0.51
**DOA**		221.14 aB ± 21.08	130.97 bA ± 96.12	221.94 aA ± 20.22	61.84 cA ± 51.00	101.51 cA ± 38.84	263.20 aB ± 88.98	110.31 cA ± 76.53	81.55 cB ± 27.37	79.59 cA ± 47.61
**10 Hy-Ol Agl**		2.81 bcA ± 2.97	0.19 aA ± 0.08	4.19 cB ± 0.25	0.48 aA ± 0.25	0.25 aA ± 0.10	1.33 abB ± 0.11	0.33 aA ± 0.18	0.62 aA ± 0.19	1.28 abB ± 0.88
**Methyl D-Ol Agl**		8.45 bdeB ± 0.60	2.11 ceA ± 0.35	13.59 aB ± 0.97	10.20 abdeB ± 2.47	7.33 deA ± 4.30	11.50 abdeB ± 1.02	11.58 abdeA ± 6.61	14.06 aA ± 3.13	10.62 abdeB ± 1.35
**D-Lig Agl**		153.30 bB ± 13.50	163.43 bcA ± 26.55	203.75 bcA ± 15.34	103.50 aA ± 21.18	89.65 aA ± 26.56	190.12 bcB ± 37.87	108.39 aA ± 38.15	70.38 adB ± 31.83	78.44 aA ± 4.17
**Dehydro Ol Agl**		1.39 bceA ± 0.19	1.59 ceA ± 0.45	1.46 ceA ± 0.14	6.44 dA ± 0.98	2.66 abcA ± 0.28	0.53 eB ± 0.12	2.19 abcA ± 1.26	2.29 abcA ± 0.96	2.95 acA ± 1.58
**Methyl Ol Agl**		1.66 bdgA ± 0.20	0.48 ceA ± 0.07	0.90 acdeA ± 0.10	0.80 aceB ± 0.36	0.73 aceB ± 0.35	1.76 dgB ± 0.14	1.30 abdA ± 0.46	0.60 eB ± 0.20	1.61 dgA ± 0.65

Significant differences in the same row are indicated with different lowercase letters (comparison among the 13 cultivars investigated in this study at the same crop season, *p* < 0.05) and with different capital letters (comparison between crop seasons for the same cultivar, *p* < 0.05). Since only the quantitative results for some of the determined compounds are included in Table 2 for 9 varieties (to contain the size of the table), the results shown herewith were considered together with those from Table 4 to carry out ANOVA analysis. Secoiridoids were quantified in terms of Oleuropein. P-Languedoc: Picholine de Languedoc.

**Table 3:** Mean ± standard deviation (mg/kg) of some of the phenolic compounds determined in the studied monovarietal olive oils.

Compound		Arbequina	Arbosana	Cornicabra	Frantoio	Hojiblanca	Koroneiki	Manzanilla	P-Languedoc	Picual
**Ol Agl**	**2012/2013**	27.66 acdA ± 6.05	23.76 adA ± 23.05	68.03 bcefA ± 8.88	30.43 adA ± 9.46	20.70 adA ± 7.56	59.22 ceA ± 17.16	46.32 abcA ± 19.21	47.30 abcA ± 8.70	39.23 acA ± 18.15
**Ol Agl IS1**		3.52 bA ± 1.66	4.84 abA ± 9.22	17.24 abdA ± 2.20	17.08 abdA ± 9.57	10.78 abdA ± 11.85	38.27 cA ± 14.05	12.24 abdA ± 12.68	25.04 cdA ± 15.61	22.15 abcdA ± 11.38
**Ol Agl IS2**		4.51 acA ± 2.44	6.36 acA ± 9.90	22.51 abdeA ± 1.54	23.22 acdeA ± 8.64	13.66 acdA ± 13.27	42.02 bA ± 15.30	15.83 acdeA ± 12.64	29.59 bdeA ± 15.22	26.75 bcdeA ± 10.89
**Ol Agl IS3**		5.56 acefA ± 1.97	4.06 abcefA ± 5.33	12.29 abcdgA ± 1.44	10.26 abcgA ± 3.78	7.77 cA ± 5.79	19.96 dA ± 7.29	10.55 abcgA ± 6.45	14.22 acdgA ± 5.92	11.97 abcgA ± 5.85
**Ol Agl IS4**		7.18 abcA ± 2.85	4.96 bcA ± 6.18	17.90 adeA ± 0.91	9.04 acA ± 3.61	8.20 acA ± 6.48	18.15 adeA ± 3.61	12.68 adeA ± 8.41	15.03 adeA ± 5.13	12.37 adeA ± 7.21
**Ol Agl IS5**		10.55 abcdA ± 2.77	7.63 bdA ± 8.17	25.11 aeA ± 1.42	11.66 abcdA ± 3.79	8.90 bcdA ± 5.69	22.39 aeA ± 6.41	16.23 abA ± 8.09	19.01 abA ± 5.30	15.84 abA ± 7.85
**Ol Agl IS6**		20.49 abcefA ± 2.78	10.25 bdA ± 7.91	19.46 aeA ± 0.95	15.67 abA ± 4.33	14.63 abA ± 6.76	26.13 cefA ± 3.46	17.66 abceA ± 7.51	21.48 aceA ± 6.75	18.37 abceA ± 6.43
**Lig Agl**		19.83 beA ± 5.57	28.32 beA ± 9.06	69.51 adfA ± 4.11	48.25 abcdA ± 10.53	25.02 ceA ± 9.23	73.66 dfA ± 16.72	47.93 abcdeA ± 19.69	64.00 adfA ± 16.25	49.48 abcdA ± 8.87
**Lig Agl IS1**		4.32 bA ± 4.52	6.83 bA ± 9.71	27.76 abcdA ± 4.98	32.91 acdeA ± 5.45	12.60 abA ± 10.79	48.91 cdeA ± 16.14	20.36 abA ± 13.48	42.05 cdeA ± 20.58	30.90 acdA ± 11.7
**Lig Agl IS2**		4.49 bdA ± 4.52	8.46 bdA ± 7.68	19.72 abcdA ± 2.34	26.41 aceA ± 4.46	12.13 abdA ± 9.71	36.49 aceA ± 12.21	17.03 abdA ± 11.05	34.32 ceA ± 14.87	24.05 acA ± 7.35
**Lig Agl IS3**		3.77 bceA ± 0.97	5.46 bceA ± 2.12	14.57 adfgA ± 2.34	9.14 abcdeA ± 3.11	4.88 ceA ± 2.01	13.26 adA ± 4.96	9.30 abceA ± 3.91	14.24 fdA ± 3.23	10.21 abceA ± 1.98
**Ol Agl**	**2013/2014**	55.36 aeB ± 6.39	21.04 bdA ± 8.39	96.88 cB ± 14.46	24.49 adA ± 14.57	35.77 abdeA ± 17.1	93.59 cB ± 9.02	54.01 aeA ± 33.17	46.63 adeA ± 10.95	51.96 aeA ± 26.81
**Ol Agl IS1**		9.16 abcdB ± 1.17	1.66 bdA ± 0.82	23.94 cB ± 2.85	9.70 abcdA ± 9.42	11.89 abcdA ± 14.31	19.27 acdB ± 1.78	14.76 abcdA ± 20.22	14.45 abcdB ± 2.69	11.10 abcdB ± 7.72
**Ol Agl IS2**		12.95 bcdeB ± 1.39	3.08 cdeA ± 1.42	29.26 aB ± 1.01	12.21 deB ± 10.52	15.92 abcdeA ± 14.99	27.74 aB ± 2.73	18.88 abdeA ± 18.98	22.97 abdeA ± 3.09	16.65 abcdeB ± 8.82
**Ol Agl IS3**		10.23 aceB ± 1.16	3.21 bceA ± 1.80	16.39 adB ± 2.68	5.62 ceB ± 4.59	9.60 abceA ± 7.79	16.65 dA ± 2.12	10.51 acdeA ± 7.52	10.64 acdeA ± 2.65	9.66 abceA ± 4.99
**Ol Agl IS4**		10.85 abdB ± 0.95	2.93 bdA ± 1.47	20.63 ceA ± 3.99	5.61 dA ± 4.41	8.84 abdA ± 6.63	20.51 ceA ± 2.85	14.27 acdeA ± 12.2	12.70 acdeA ± 3.81	10.93 abcdA ± 6.13
**Ol Agl IS5**		15.25 abeB ± 2.07	6.04 bA ± 2.24	32.83 cdeB ± 4.91	8.68 abA ± 6.39	11.74 abA ± 7.25	29.00 deB ± 4.14	19.12 aeA ± 12.08	16.99 aeA ± 4.18	16.16 aeA ± 9.53
**Ol Agl IS6**		19.23 acdA ± 1.41	10.97 bcA ± 3.67	24.31 adB ± 2.25	12.03 cA ± 6.25	18.31 abcA ± 7.54	26.65 dA ± 1.80	18.98 abcdA ± 11.01	21.19 adA ± 1.74	16.44 abcA ± 6.14
**Lig Agl**		53.97 adeB ± 4.39	18.91 beB ± 2.19	93.88 cB ± 6.37	34.84 abdeB ± 8.74	41.09 abdeA ± 18.11	66.09 dB ± 15.78	65.63 cdA ± 37.07	46.29 adeB ± 14.40	47.39 adeA ± 6.08
**Lig Agl IS1**		18.67 adB ± 2.35	3.41 bA ± 0.44	38.32 cdB ± 3.94	21.11 adB ± 11.25	11.62 abA ± 8.69	24.97 acdB ± 4.13	22.18 adA ± 18.25	27.03 dA ± 12.37	16.83 abdB ± 2.76
**Lig Agl IS2**		18.17 adB ± 2.48	3.72 bA ± 0.53	28.35 cB ± 1.91	17.57 acdB ± 6.29	13.17 adA ± 8.72	17.84 acdB ± 3.23	19.05 acdA ± 12.52	21.19 acB ± 7.08	13.23 adB ± 2.54
**Lig Agl IS3**		9.01 aB ± 2.23	3.29 aB ± 0.42	20.63 bcB ± 1.32	5.84 aB ± 1.79	7.57 aA ± 3.20	19.36 cA ± 16.38	12.73 abcA ± 7.28	9.6 3aB ± 3.03	9.53 abA ± 2.07

Significant differences in the same row are indicated with different lowercase letters (comparison among the 13 cultivars at the same crop season, *p* < 0.05) and with different capital letters (comparison between crop seasons for the same cultivar, *p* < 0.05). Quantitative results for some compounds from 9 varieties are included in this table (to contain its size); therefore, the concentration levels shown herewith were treated together with those from Table 4 to carry out ANOVA analysis. Secoiridoids were quantified in terms of Oleuropein. P-Languedoc: Picholine de Languedoc.

**Table 4:** Mean ± standard deviation (mg/kg) of some of the phenolic compounds determined in the studied monovarietal olive oils.

Compound		Picholine Marocaine	Dahbia	Haouzia	Menara
**D-Ald-D EA**	**2012/2013**	1.65 aA ± 3.14	0.26 aA ± 0.12	0.83 aA ± 0.87	1.69 aA ± 2.60
**Desoxy-EA**		9.60 ac ± 4.50	0.40 a ± 0.13	12.40 acd ± 13.39	11.15 acd ± 18.56
**Hy-EA**		0.19 a ± 0.13	0.23 a ± 0.05	0.15 a ± 0.06	0.12 a ± 0.05
**EA**		20.69 aA ± 5.49	5.73 dA ± 1.44	17.35 adA ± 5.04	33.68 acA ± 12.23
**Hy D-Ol Agl**		0.31 bA ± 0.22	0.78 abA ± 0.42	0.20 bA ± 0.15	0.35 abA ± 0.40
**DOA**		133.66 bA ± 114.84	3.15 dA ± 1.19	18.94 bA ± 14.31	17.00 bA ± 8.64
**10 Hy-Ol Agl**		0.36 aA ± 0.07	0.37 aA ± 0.08	0.17 aA ± 0.09	0.17 aA ± 0.05
**Methyl D-Ol Agl**		10.96 cdgA ± 2.34	10.25 abcgA ± 1.63	20.46 eA ± 4.83	28.81 fA ± 8.50
**D-Lig Agl**		84.50 fA ± 47.17	0.46 deA ± 0.04	2.78 deA ± 1.45	2.41 eA ± 1.07
**Dehydro Ol Agl**		2.30 aA ± 1.29	15.75 dA ± 2.32	3.59 bceA ± 2.55	5.45 eA ± 1.82
**Methyl Ol Agl**		1.61 aA ± 1.00	2.05 abcdA ± 0.23	3.02 dA ± 1.80	1.93 abdA ± 0.55
**Ol Agl**		45.79 abcA ± 17.62	8.73 dA ± 1.52	92.35 efA ± 36.41	98.33 fA ± 34.50
**Ol Agl IS1**		15.42 abdA ± 6.11	4.59 bA ± 3.28	26.59 acdA ± 19.53	25.80 acdA ± 11.15
**Ol Agl IS2**		23.34 acdeA ± 7.86	8.95 cA ± 5.45	36.03 bdeA ± 19.49	40.59 beA ± 15.59
**Ol Agl IS3**		10.65 ceA ± 4.46	5.19 cfA ± 2.05	20.60 dgA ± 10.61	20.32 dgA ± 9.00
**Ol Agl IS4**		14.04 adeA ± 5.87	1.05 cA ± 0.60	21.89 deA ± 12.89	21.99 eA ± 9.68
**Ol Agl IS5**		18.07 abA ± 6.83	1.75 dA ± 0.45	32.13 eA ± 14.04	33.92 eA ± 15.93
**Ol Agl IS6**		18.22 abceA ± 4.57	2.78 dA ± 0.81	27.69 efA ± 9.88	31.83 fA ± 7.31
**Lig Agl**		42.62 abcegA ± 15.20	15.08 eA ± 2.70	95.84 fgA ± 26.78	113.01 gA ± 48.67
**Lig Agl IS1**		18.55 abA ± 5.79	9.74 bA ± 2.04	33.34 acdeA ± 12.23	53.91 eA ± 29.04
**Lig Agl IS2**		16.10 abdA ± 4.76	7.08 dA ± 2.85	26.68 aceA ± 13.61	42.72 eA ± 13.16
**Lig Agl IS3**		9.38 abceA ± 3.75	4.25 eA ± 5.96	21.41 fgA ± 7.65	22.54 gA ± 7.56
**D-Ald-D EA**	**2013/2014**	1.72 aA ± 2.07	0.33 aA ± 0.05	7.50 aA ± 9.24	15.58 aA ± 17.95
**Desoxy-EA**		18.11 abB ± 2.88	0.53 dA ± 0.14	7.80 abA ± 2.50	10.70 abA ± 6.33
**Hy-EA**		0.20 aA ± 0.12	0.10 aB ± 0.03	0.47 aceA ± 0.46	0.41 aceB ± 0.08
**EA**		14.35 dB ± 3.35	22.26 abdB ± 1.63	55.48 abcB ± 10.45	51.04 abA ± 23.13
**Hy D-Ol Agl**		0.18 bA ± 0.09	0.10 bB ± 0.02	1.04 bA ± 1.46	0.76 bA ± 0.27
**DOA**		39.58 cB ± 9.24	58.06 cB ± 8.98	87.24 cB ± 54.4	70.68 cB ± 21.18
**10 Hy-Ol Agl**		0.60 aB ± 0.08	0.14 aB ± 0.06	1.27 abA ± 1.12	0.87 aB ± 0.26
**Methyl D-Ol Agl**		12.92 aB ± 1.46	7.45 deB ± 0.44	12.84 abdeB ± 0.74	10.91 abdeB ± 2.28
**D-Lig Agl**		30.68 dB ± 9.99	93.73 aB ± 10.60	57.94 adA ± 55.28	61.62 adB ± 21.87
**Dehydro Ol Agl**		2.87 acA ± 0.60	6.29 dB ± 0.63	3.46 aA ± 1.09	3.54 aB ± 0.67
**Methyl Ol Agl**		0.88 aceB ± 0.47	2.65 fB ± 0.35	2.23 fgA ± 0.58	2.75 fB ± 0.38
**Ol Agl**		35.20 abdA ± 7.56	26.12 abdB ± 0.42	63.47 eA ± 12.68	41.56 abdeB ± 10.47
**Ol Agl IS1**		19.16 acdA ± 6.21	4.28 dA ± 0.43	23.29 acA ± 3.67	16.22 acdA ± 8.80
**Ol Agl IS2**		28.84 aA ± 6.80	7.31 eA ± 1.05	31.20 aA ± 3.31	23.85 abdeB ± 10.83
**Ol Agl IS3**		11.02 acdeA ± 2.52	5.17 eA ± 0.52	16.97 adA ± 3.22	12.51 acdeA ± 4.85
**Ol Agl IS4**		16.02 aceA ± 3.69	3.73 dB ± 0.38	16.80 aceA ± 2.93	13.36 acdeA ± 5.23
**Ol Agl IS5**		15.80 aeA ± 3.35	8.32 abB ± 0.71	23.45 eA ± 3.38	18.12 aeB ± 5.06
**Ol Agl IS6**		20.27 adA ± 2.82	14.75 abcB ± 0.45	21.90 abdA ± 1.58	16.89 abcB ± 3.77
**Lig Agl**		35.94 beA ± 3.67	33.13 beB ± 0.96	51.14 adeB ± 22.8	43.75 abdeB ± 5.34
**Lig Agl IS1**		20.23 adA ± 2.94	10.95 abdA ± 1.01	26.80 acdA ± 7.07	21.27 adB ± 6.22
**Lig Agl IS2**		19.16 acdA ± 4.03	9.28 dA ± 1.11	20.06 acA ± 2.04	17.53 adB ± 4.93
**Lig Agl IS3**		7.93 aA ± 2.07	6.36 aA ± 1.08	10.39 abcB ± 3.99	9.09 abB ± 1.08

Significant differences in the same row are indicated with different lowercase letters (comparison among the 13 cultivars at the same crop season, *p* < 0.05) and with different capital letters (comparison between crop seasons for the same cultivar, *p* < 0.05). Quantitative results for some compounds determined in 4 varieties from those 13 selected for this study are included in this table (to contain its size); therefore, the results shown herewith were treated together with those from Table 2 and Table 3 to carry out ANOVA analysis. Secoiridoids were quantified in terms of Oleuropein.

**Table 5:** Data about planting date and distance of the studied cultivars, number of samples per cultivar and per crop season, quality parameters of the obtained oils (mean ± standard deviation of free fatty acids (FAAs), peroxide values (PV) and ultraviolet absorbance at 232 (*K*_232_) and 270 nm (*K*_270_)), and pedoclimatic conditions of the experimental orchard during the study period.

Orchard Characteristics	Arbequina	Arbosana	Cornicabra	Frantoio	Hojiblanca	Koroneiki	Manzanilla	Picholine-Languedoc	Picual	Dahbia	Haouzia	Menara	Picholine Marocaine
**Planting date**	2001		2000				2001	2000	2001	2000			40 years ago
**Planting distances**	7 × 5 **(between rows × between trees)**												10 × 10
**2012/2013 (total = 105)**	6	7	6	9	6	9	10	10	10	7	5	6	14
**2013/2014 (total = 98)**	10	8	5	7	7	9	7	10	8	4	6	7	10
**Oils quality parameters**													
**FFAs (% oleic acid)**	0.25 ± 0.04	0.22 ± 0.05	0.19 ± 0.03	0.22 ± 0.06	0.20 ± 0.05	0.23 ± 0.03	0.21 ± 0.05	0.21 ± 0.04	0.20 ± 0.05	0.20 ± 0.06	0.20 ± 0.05	0.24 ± 0.04	0.24 ± 0.03
**PV (meq O_2_/kg)**	4.99 ± 0.91	5.12 ± 0.77	5.01 ± 0.87	5.18 ± 0.76	5.40 ± 0.56	5.07 ± 0.86	5.04 ± 0.83	5.02 ± 0.88	5.30 ± 0.96	4.82 ± 0.82	5.02 ± 0.92	5.04 ± 0.80	5.23 ± 0.92
***K*_232_**	0.94 ± 0.11	0.90 ± 0.15	0.92 ± 0.10	0.95 ± 0.13	0.95 ± 0.14	0.94 ± 0.13	0.95 ± 0.12	0.95 ± 0.12	0.94 ± 0.13	0.95 ± 0.12	0.93 ± 0.15	0.98 ± 0.10	0.95 ± 0.12
***K*_270_**	0.09 ± 0.02	0.10 ± 0.03	0.11 ± 0.02	0.10 ± 0.03	0.11 ± 0.03	0.13 ± 0.01	0.11 ± 0.03	0.12 ± 0.03	0.12 ± 0.02	0.12 ± 0.03	0.11 ± 0.03	0.12 ± 0.02	0.12 ± 0.01
**Quality category**	Extra-virgin												
**Site climatic data**													
**Month**		**January**	**February**	**March**	**April**	**May**	**June**	**July**	**August**	**September**	**October**	**November**	**December**
**Average maximum temperatures (°C)**	2012	16.30	14.90	20.90	19.60	29.90	32.90	35.40	35.90	30.70	25.48	16.67	15.25
	2013	15.51	15.52	15.59	22.70	25.19	32.30	37.53	37.97	30.83	26.80	19.00	16.70
**Average minimum temperatures (°C)**	2012	4.20	2.90	6.80	8.40	14.10	17.40	18.40	18.90	16.80	12.52	16.67	15.25
	2013	9.29	8.84	9.96	15.50	16.81	22.63	29.23	29.67	22.89	13.90	8.30	5.40
**Total rainfall (mm)**	2012	35.50	13.00	20.00	105.00	12.00	0.00	0.00	0.00	0.00	136.50	156.00	56.00
	2013	98.50	76.50	177.00	45.50	32.00	0.00	0.00	0.00	32.50	8.00	89.00	42.00
**Soil characteristics**													
	**Depth (cm)**	**pH (H_2_O)**	**Clay (%)**	**Slit (%)**	**Sand (%)**	**CaCO_3_ (%)**	**Humus (%)**	**Al–P_2_O_5_ (mg/kg)**	**Al–K_2_O (mg/kg)**	**Copper (mg/kg)**	**Zinc (mg/kg)**	**Iron (mg/kg)**	**Manganese (mg/kg)**
**Horizon 1**	0–30	8.50	68.30	23.80	7.90	1.70	2.59	12.00	493.00	2.91	0.36	7.98	591.00
**Horizon 2**	30–60	8.60	64.50	25.40	10.10	2.80	1.87	2.00	246.00	1.00	0.13	7.51	417.00

## Supplementary Materials

Supplementary materials can be found at www.mdpi.com/1422-0067/18/1/52/s1.
