# Supplementary material for: In-Depth Two-Year Study of Phenolic Profile Variability among Olive Oils from Autochthonous and Mediterranean Varieties in Morocco, as Revealed by a LC-MS Chemometric Profiling Approach
Source: Int J Mol Sci. 2016 Dec 28;18(1):52. doi: 10.3390/ijms18010052 (PMC5297687; doi:10.3390/ijms18010052)
Supplement: Supplementary file 1 [file ijms-18-00052-s001.pdf]

Supplementary Materials: In-Depth Two-Year Study of Phenolic Profile Variability among Olive Oils from Autochthonous and Mediterranean Varieties in Morocco, as Revealed by a LC-MS Chemometric Profiling Approach

Aadil Bajoub, Santiago Medina-Rodríguez, Lucía Olmo-García, El Amine Ajal, Romina P. Monasterio, Hafida Hanine, Alberto Fernández-Gutiérrez and Alegría Carrasco-Pancorbo

Table S1. Classification matrix, according to LDA, for the varietal origin discrimination between VOOs from "Picholine Marocaine" and Mediterranean cultivars (varietal origin discriminant Model 1).

Classification	Arbequina	Arbosana	Cornicabra	Frantoio	Hojiblanca	Koroneiki	Manzanilla	Picholine Marocaine	Picholine de Languedoc	Picual	Total	% Correct
**Arbequina**	16	0	0	0	0	0	0	0	0	0	16	100.00%
**Arbosana**	1	13	0	0	0	0	0	1	0	0	15	86.67%
**Cornicabra**	0	0	11	0	0	0	0	0	0	0	11	100.00%
**Frantoio**	0	0	0	15	0	0	0	1	0	0	16	93.75%
**Hojiblanca**	0	0	0	0	13	0	0	0	0	0	13	100.00%
**Koroneiki**	0	0	0	0	0	18	0	0	0	0	18	100.00%
**Manzanilla**	0	0	0	0	0	0	17	0	0	0	17	100.00%
**Picholine Marocaine**	0	0	0	0	0	0	0	24	0	0	24	100.00%
**Picholine de Languedoc**	0	0	0	0	0	0	0	3	17	0	20	85.00%
**Picual**	0	0	0	0	0	0	0	1	1	16	18	88.89%
**Total**	**17**	**13**	**11**	**15**	**13**	**18**	**17**	**30**	**18**	**16**	**168**	**95.24%**
**Cross-Validation**	**Arbequina**	**Arbosana**	**Cornicabra**	**Frantoio**	**Hojiblanca**	**Koroneiki**	**Manzanilla**	**Picholine Marocaine**	**Picholine de Languedoc**	**Picual**	**Total**	**% Correct**
**Arbequina**	14	0	1	1	0	0	0	0	0	0	16	87.50%
**Arbosana**	1	13	0	0	0	0	0	1	0	0	15	86.67%
**Cornicabra**	0	0	11	0	0	0	0	0	0	0	11	100.00%
**Frantoio**	0	0	0	15	0	0	0	1	0	0	16	93.75%
**Hojiblanca**	0	0	0	0	13	0	0	0	0	0	13	100.00%
**Koroneiki**	0	0	0	0	1	17	0	0	0	0	18	94.44%
**Manzanilla**	0	0	0	0	0	0	17	0	0	0	17	100.00%
**Picholine Marocaine**	0	0	0	0	0	0	0	24	0	0	24	100.00%
**Picholine de Languedoc**	1	0	0	0	0	0	0	3	16	0	20	80.00%
**Picual**	0	0	0	0	0	0	0	1	1	16	18	88.89%
**Total**	**16**	**13**	**12**	**16**	**14**	**17**	**17**	**30**	**17**	**16**	**168**	**92.86%**

Table S2. Classification matrix, according to LDA, for the botanical origin discrimination between VOOs from "Picholine Marocaine" and Moroccan cultivars (varietal origin discriminant Model 2).

Classification	Dahbia	Haouzia	Menara	Picholine Marocaine	Total	% Correct
**Dahbia**	11	0	0	0	11	100.00%
**Haouzia**	0	11	0	0	11	100.00%
**Menara**	0	0	12	0	12	100.00%
**Picholine Marocaine**	0	0	0	24	24	100.00%
**Total**	**11**	**11**	**12**	**24**	**58**	**100.00%**
**Cross-validation**	**Dahbia**	**Haouzia**	**Menara**	**Picholine Marocaine**	**Total**	**% Correct**
**Dahbia**	11	0	0	0	11	100.00%
**Haouzia**	0	9	1	1	11	81.82%
**Menara**	0	1	7	4	12	58.33%
**Picholine Marocaine**	0	0	1	23	24	95.83%
**Total**	**11**	**10**	**9**	**28**	**58**	**86.21%**

Table S3. Classification matrix, according to LDA, for the botanical origin discrimination between VOOs obtained from Mediterranean and Moroccan cultivars (varietal origin discriminant Model 3).

Classification	Arbequina	Arbosana	Cornicabra	Dahbia	Frantoio	Hojiblanca	Haouzia	Koroneiki	Manzanilla	Menara	Picholine de Languedoc	Picual	Total	% Correct
**Arbequina**	15	0	0	0	0	0	0	0	0	0	1	0	16	93.75%
**Arbosana**	1	13	0	0	0	0	0	0	0	0	1	0	15	86.67%
**Cornicabra**	0	0	11	0	0	0	0	0	0	0	0	0	11	100.00%
**Dahbia**	0	0	0	11	0	0	0	0	0	0	0	0	11	100.00%
**Frantoio**	0	0	0	0	15	0	0	0	0	0	1	0	16	93.75%
**Hojiblanca**	0	0	0	0	0	13	0	0	0	0	0	0	13	100.00%
**Haouzia**	0	0	1	0	0	0	9	0	0	1	0	0	11	81.82%
**Koroneiki**	0	0	0	0	0	0	0	17	0	0	0	1	18	94.44%
**Manzanilla**	0	2	0	0	0	0	0	0	15	0	0	0	17	88.24%
**Menara**	0	0	0	0	0	0	1	0	0	11	0	0	12	91.67%
**Picholine de Languedoc**	0	0	0	0	1	0	0	0	0	0	19	0	20	95.00%
**Picual**	0	0	0	0	0	0	0	0	0	0	1	17	18	94.44%
**Total**	**16**	**15**	**12**	**11**	**16**	**13**	**10**	**17**	**15**	**12**	**23**	**18**	**178**	**93.26%**
**Cross-validation**	**Arbequina**	**Arbosana**	**Cornicabra**	**Dahbia**	**Frantoio**	**Hojiblanca**	**Haouzia**	**Koroneiki**	**Manzanilla**	**Menara**	**Picholine de Languedoc**	**Picual**	**Total**	**% Correct**
**Arbequina**	15	0	0	0	0	0	0	0	0	0	1	0	16	93.75%
**Arbosana**	1	11	0	0	0	0	0	0	0	1	1	1	15	73.33%
**Cornicabra**	0	0	11	0	0	0	0	0	0	0	0	0	11	100.00%
**Dahbia**	0	0	0	11	0	0	0	0	0	0	0	0	11	100.00%
**Frantoio**	0	0	0	0	14	0	0	0	0	1	1	0	16	87.50%
**Hojiblanca**	0	0	0	0	0	13	0	0	0	0	0	0	13	100.00%
**Haouzia**	0	0	1	0	0	0	6	0	1	3	0	0	11	54.55%
**Koroneiki**	0	0	0	0	0	0	0	17	0	0	0	1	18	94.44%
**Manzanilla**	0	2	0	0	0	0	0	0	15	0	0	0	17	88.24%
**Menara**	0	0	0	0	1	0	4	0	0	6	1	0	12	50.00%
**Picholine de Languedoc**	0	0	0	0	1	0	1	1	0	1	16	0	20	80.00%
**Picual**	0	0	0	0	0	0	1	0	0	0	1	16	18	88.89%
**Total**	**16**	**13**	**12**	**11**	**16**	**13**	**12**	**18**	**16**	**12**	**21**	**18**	**178**	**84.83%**
